# *Cryptosporidium* spp. Infection and Genotype Identification in Pre-Weaned and Post-Weaned Calves in Yunnan Province, China

**DOI:** 10.3390/ani14131907

**Published:** 2024-06-27

**Authors:** Meng-Ling Deng, Zhao-Jun Heng, Liu-Jia Li, Jian-Fa Yang, Jun-Jun He, Feng-Cai Zou, Fan-Fan Shu

**Affiliations:** 1 Faculty of Animal Science and Technology, Yunnan Agricultural University, Kunming 650201, China; merlideng@126.com (M.-L.D.); jsc315@163.com (J.-F.Y.); 2 Key Laboratory of Veterinary Public Health of Yunnan Province, College of Veterinary Medicine, Yunnan Agricultural University, Kunming 650201, China; hzhaoj123@126.com (Z.-J.H.); hejunjun617@163.com (J.-J.H.); 3 College of Agriculture and Biological Science, Dali University, Dali 671003, China; liliujia2007@163.com

**Keywords:** *Cryptosporidium* spp., Holstein calves, occurrence, subtype, public health

## Abstract

**Simple Summary:**

Cryptosporidiosis is a significant zoonotic parasitic disease, and the infected animals can be a substantial source of human infection. However, there is limited research on the occurrence of *Cryptosporidium* spp. in calves in Yunnan Province. Therefore, the current study aimed to investigate the species and genotypes of *Cryptosporidium* spp. in pre-weaned and post-weaned Holstein calves, and to reveal their potential zoonotic risk in Yunnan Province, China. The findings revealed that the infection rate of *Cryptosporidium* spp. in Holstein calves was 32.9% (164/498), and four different *Cryptosporidium* species were involved, namely *C. bovis*, *C. parvum*, *C. ryanae* and *C. andersoni*. The results highlighted a high occurrence and rich genetic diversity of *Cryptosporidium* spp. in pre-weaned and post-weaned Holstein calves in Yunnan. Notably, the detection of two zoonotic subtypes, IIdA18G1 and IIdA19G1, of *C. parvum* suggested that these calves were crucial to the spread of zoonosis in Yunnan Province.

**Abstract:**

Background: *Cryptosporidium* is a globally distributed zoonotic protozoan parasite in humans and animals. Infection is widespread in dairy cattle, especially in calves, resulting in neonatal enteritis, production losses and high mortality. However, the occurrence of *Cryptosporidium* spp. in pre- and post-weaned calves in Yunnan Province remains unclear. Methods: We collected 498 fecal samples from Holstein calves on 10 different farms in four regions of Yunnan Province. Nested PCR and DNA sequencing were used to determine the infection, species and genotypes of *Cryptosporidium* spp. in these animals. Results: The overall occurrence of *Cryptosporidium* spp. in Holstein calves was 32.9% (164/498), and the prevalence in pre- and post-weaned calves was 33.5% (106/316) and 31.9% (58/182), respectively. Four *Cryptosporidium* species were identified in these animals, namely *C. bovis* (*n* = 119), *C. parvum* (*n* = 23), *C. ryanae* (*n* = 20) and *C. andersoni* (*n* = 2). Based on sequencing analysis of the 60 kDa glycoprotein gene of *C. bovis*, *C. parvum* and *C. ryanae*, six subtypes of *C. bovis* (XXVIe, XXVIb, XXVIf, XXVIa XXVIc and XXVId), two subtypes of *C. parvum* (IIdA19G1 and IIdA18G1) and four subtypes of *C. ryanae* (XXIf, XXId, XXIe and XXIg) were identified. Conclusions: These results provide essential information to understand the infection rate, species diversity and genetic structure of *Cryptosporidium* spp. populations in Holstein pre-weaned and post-weaned calves in Yunnan Province. Further, the presence of IIdA18G1 and IIdA19G1 in *C. parvum* implies significant animal and public health concerns, which requires greater attention and more preventive measures.

## 1. Introduction

*Cryptosporidium* spp. are recognized as globally distributed pathogens responsible for large food- and water-borne outbreaks of gastroenteritis in various vertebrates [1]. These species are most likely to infect children and young animals, causing diarrhea-related fatalities and gastrointestinal illness [2]. Cryptosporidiosis is the leading cause of diarrhea and diarrhea-related deaths among children in low- and middle-income countries [2]. In China, human cryptosporidiosis has been reported in more than 25 provinces [3] and is responsible for 1.4% to 10.4% of diarrhea episodes [4]. Human infection with *Cryptosporidium* occurs through consumption of contaminated food or water, environmental exposure and direct contact with infected humans and animals [1]. Infected cattle, especially pre- and post-weaned calves, are major reservoirs for human infections [5,6]. *Cryptosporidium* infection in animals can cause severe diarrhea, weight loss and even death in young animals in the short term, with a mortality rate of 44.4% in pre-weaning calves. After recovery, many animals also experience long-term weight gain reduction and growth retardation [7,8,9,10], resulting in significant production losses and economic burden. In recent years, many outbreaks of cryptosporidiosis in cattle have been reported in China, all with long-term adverse effects on public health and the livestock industry [11,12,13,14].

To date, more than 44 species of *Cryptosporidium* spp. and at least 120 genotypes have been described worldwide [15]. Among them, *C. parvum*, *C. bovis*, *C. ryanae* and *C. andersoni* are routinely detected in dairy cattle [16]. Since the first microscopic examination analysis of *Cryptosporidium* in dairy cattle was reported in Lanzhou, China, in 1986 [17], 24 provinces, autonomous regions and municipalities have seen *Cryptosporidium* infection in dairy cattle [18]. Many studies have demonstrated an age-related distribution of the four common species in cattle. In most industrialized countries, *C. parvum* is the major cause of diarrhea in newborn calves during the first two weeks after birth, and is also responsible for most cases of human cryptosporidiosis. *C. bovis* is the dominant species in calves between 3 and 9 weeks of age, with a peak occurrence in calves at 6 weeks of age. *C. ryanae* can be detected in 4- to 8-week-old calves [19], and *C. andersoni* is usually found in adult cattle with poor weight gain and milk yield [20]. However, the prevalence pattern of *Cryptosporidium* spp. appears to vary between different host species and geographical regions of the world [21,22].

Several genetic typing tools have been developed to better understand the molecular epidemiology of *Cryptosporidium* spp. The 60 kDa glycoprotein (*gp*60) gene is the most commonly used genetic marker for typing *C. parvum*, *C. bovis* and *C. ryanae* due to its high degree of genetic variation and relevance to parasite biology [23,24]. Nearly 20 different *gp*60 subtypes have been observed in *C. parvum*, with Ⅱa and Ⅱd being the dominant subtype families, and their geographic distribution is variable [25]. The IIa family is mainly prevalent in developed countries, while IId is a dominant subtype family in China and some developing countries [18]. Six subtype families, XXVIa to XXVIf, have been identified in *C. bovis*, and infection with XXVId correlates with moderate diarrhea in dairy cattle [26]. Eight subtypes of *C. ryanae*, XXIa to XXIh, have been identified, showing possible host adaptability, with geographical differences in the distribution of dairy cattle [27]. For *C. andersoni*, multilocus sequence typing (MLST) is commonly used, and at least 10 subtypes have been reported in cattle in China [28].

Dairy calves before and after weaning are important hosts for *Cryptosporidium*. Bovine cryptosporidiosis has been identified as a significant contributor to neonatal diarrhea and financial losses on dairy farms [29]. Among these pathogens, at least 10 species (i.e., *C. bovis*, *C. andersoni*, *C. ryanae*, *C. parvum*, *C. xiaoi*, *C. ubiquitum*, *C. meleagridis*, *C. hominis*, *C. tyzzeri* and *C. serpentis*) have been reported in cattle in several provinces in China [11,12,14,16,19,30,31,32,33,34,35,36,37,38,39,40]. In recent years, Yunnan has had the highest number of cattle in China. The monsoon climate of Yunnan provides a high-quality environment for cattle breeding. However, it also facilitates the survival and spread of *Cryptosporidium*. Although there have been some studies on *Cryptosporidium* spp. in cattle in Yunnan, most of them have focused on adult cattle [35,41], and there are few data on *Cryptosporidium* infection in pre-weaned and post-weaned calves. Therefore, more research is needed to fully understand the prevalence, distribution and impact of *Cryptosporidium* in calves in Yunnan Province.

The aim of this study was to examine the prevalence and molecular characteristics of *Cryptosporidium* spp. in pre-weaned (aged 0–60 days) and post-weaned (aged 61–180 days) calves from 10 farms in four regions of Yunnan Province, and to assess their zoonotic potential.

## 2. Materials and Methods

### 2.1. Specimen Collection

**Figure 1:**
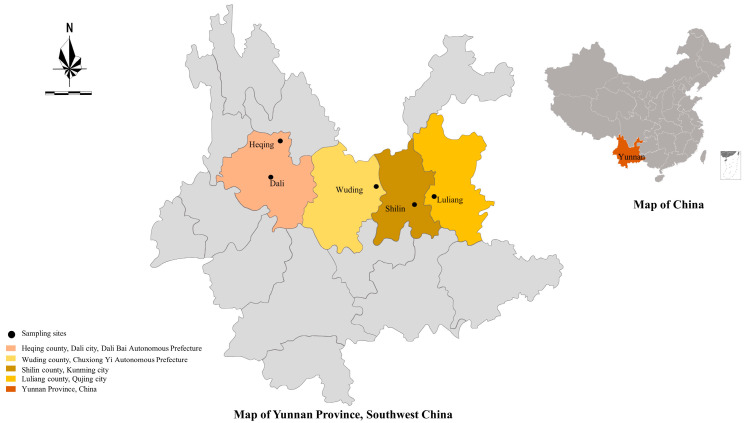
Map of sampling sites for Holstein calves in Yunnan Province, China.

From July 2021 to June 2023, a total of 498 fresh fecal samples were collected directly from the rectum of pre- and post-weaned calves on 10 different farms located in Dali (three dairy cattle farms in Heqing County, and three in Dali City), Kunming (one dairy cattle farm in Shilin County), Qujing *(*two dairy farms in Luliang County) and Chuxiong (one dairy cattle farm in Wuding County), in Yunnan Province (Figure 1). All four regions experience a subtropical monsoon climate throughout the year, with a relative humidity ranging from 70% to 80% and an annual average temperature of between 16.0 and 20.0 °C. To avoid cross-contamination, the fecal samples from each farm were collected separately using sterile plastic gloves. The date of sampling, distribution, age and gender were recorded for each animal. The cattle were categorized into pre-weaned (aged 0–60 days) calves and post-weaned (aged 61–180 days) calves according to the Technical Specification for Standardized Scale Breeding and Production of Dairy Cows (Trial) issued by the Ministry of Agriculture of the People’s Republic of China. All fecal samples were stored at 4 °C, or transferred immediately to the laboratory for DNA extraction.

### 2.2. DNA Extraction and PCR Amplification

Genomic DNA was extracted by using the E.Z.N.A.R^®^ Stool DNA Kit (Omega Bio-tek Inc., Norcross, GA, USA) according to the manufacturer’s instructions. Before DNA extraction, the stored feces were washed with distilled water and centrifuged at 3000× *g* for 3 min. Then, 250 mg of each washed fecal sample was used for DNA extraction and the DNA was stored at −20 °C for subsequent experiments.

For the detection of *Cryptosporidium* spp., an 830-bp fragment of the small subunit rRNA (*SSU* rRNA) gene was amplified by nested PCR [42]. Simultaneously, the DNA of *C. parvum*, *C. bovis* and *C. ryane* wassubtyped by amplification of the *gp60* gene according to previous studies [26,27,43]. All *C. andersoni*-positive samples were subtyped by analyzing four minisatellite/microsatellite targets (MS1, MS2, MS3, MS16) [28]. The primers, annealing temperatures and fragment lengths of the nested PCR are listed in Table S1 (Additional files: Table S1).

The nested PCR amplification was performed in a 25 μL reaction system containing 2 μL of genomic DNA for the primary PCR or 2 μL of the first PCR amplification product for the secondary PCR, 2.5 μL 10 × PCR buffer, 200 μM of each dNTP, 1 unit of *r-Taq* DNA polymerase (TaKaRa Shuzo Co., Ltd., Dalian, China), 0.4 μM of each primer and 2 mM MgCl_2_. The secondary PCR products were visualized by electrophoresis in 1% agarose gel and photographed by using a gel imaging system. Positive PCR products were subjected to DNA sequencing for species/subtype identification.

### 2.3. Sequence Analysis and Phylogenetic Tree

All positive secondary nested PCR products were sent to Sangon Biotech (Kunming, China) for bidirectional sequencing on an ABI 3730XL sequencer (Applied Biosystems, Foster City, CA, USA). The sequences were aligned and edited using ChromasPro 2.1.10.1 (http://technelysium.com.au/wp/chromaspro/, accessed on 5 June 2024). The consensus sequences were then searched against the GenBank database using the Basic Local Alignment Search Tool (https://blast.ncbi.nlm.nih.gov/Blast.cgi, accessed on 5 June 2024) to identify the species/genotypes present. The phylogenetic tree of *Cryptosporidium* spp. was constructed by using the maximum likelihood method in MEGA 11 (https://www.megasoftware.net/, accessed on 5 June 2024), and the reliability of the phylogenetic tree was assessed using the general time-reversible model and bootstrapping with 1000 replicates.

### 2.4. Statistical Analysis

The *p*-value, odds ratio (OR) and 95% confidence interval (95%CI) were calculated using SPSS 24.0 software (IBM Corp., Armonk, NY, USA), and the differences in *Cryptosporidium* spp. infection in Holstein cattle at different locations, genders and ages were analyzed. Differences were considered significant when the *p*-value ≤ 0.05. ORs with 95%CIs were used to assess the strength of the risk factors.

## 3. Results

### 3.1. Prevalence of Cryptosporidium spp.

Of the 498 fecal samples examined, 32.9% (*n* = 164) were positive for *Cryptosporidium* spp. based on *SSU* rRNA gene sequence analysis. *Cryptosporidium* prevalence was significantly influenced by region. Among the four regions, the infection rate ranged from 12.7% to 45.3%, with the prevalence of *Cryptosporidium* spp. being highest in Kunming (45.3%, 24/53), followed by Dali (38.5%, 120/312), Chuxiong (26.1%, 6/23) and Qujing (12.7%, 14/110) (Table 1). Regarding gender, the infection rate of *Cryptosporidium* spp. was higher in males (34.9%, 37/106) than in females (32.4%, 127/392). Regarding weaning status, the infection rate in post-weaned calves (31.9%, 58/182) was lower than that in pre-weaned calves (33.5%, 106/316). The Chi-squared test showed no significant difference in the prevalence of *Cryptosporidium* infection between age or gender groups (*p* = 0.63 and 0.70, respectively) (Table 1).

### 3.2. Genotyping of Cryptosporidium spp.

**Figure 2:**
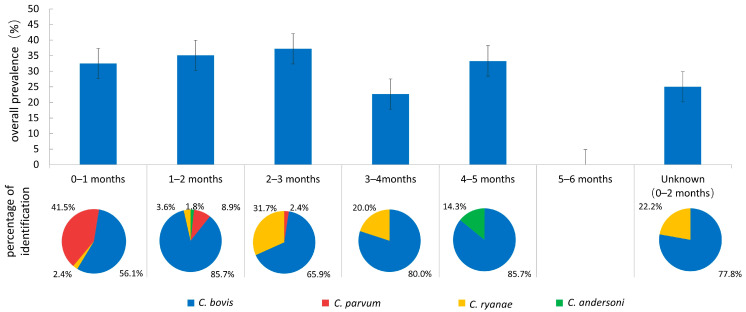
Prevalence of *Cryptosporidium* spp. and percentage of *Cryptosporidium* species identified in different age groups.

Genotyping revealed four different *Cryptosporidium* species: *C. bovis* (119/164, 72.6%), *C. parvum* (23/164, 14.6%), *C. ryanae* (20/164, 12.2%) and *C. andersoni* (2/164, 1.2%) (Table 2). *C. bovis* accounted for 72.6% (119/164) of the total and was found in all four areas, indicating its dominance and wide distribution in Yunnan Province; *C. parvum* was detected in all areas except Chuxiong; *C. ryanae* was found in Dali and Qujing; and *C. andersoni* was detected only in Dali. All four *Cryptosporidium* species were detected in Holstein cattle both before and after weaning, but the prevalence of the different species found showed different patterns when considering the age classes (Figure 2). *C. bovis* was the most dominant species in both pre-weaned (73.6%, 78/106) and post-weaned calves (70.7%, 41/58), and was found in all age groups except calves > 5 months, with the highest infection rate observed in 1–2 months. *C. parvum* infection was detected exclusively in calves ≤ 3 months of age and showed an overwhelming prevalence in suckling calves younger than 1 month. The prevalence peaked at 0–1 month of age and declined rapidly thereafter. *C. ryanae* was detected at 0–4 months of age and was found primarily in post-weaned calves. *C. andersoni* infection was sporadically detected at 1–2 months and 4–5 months of age.

**Figure 3:**
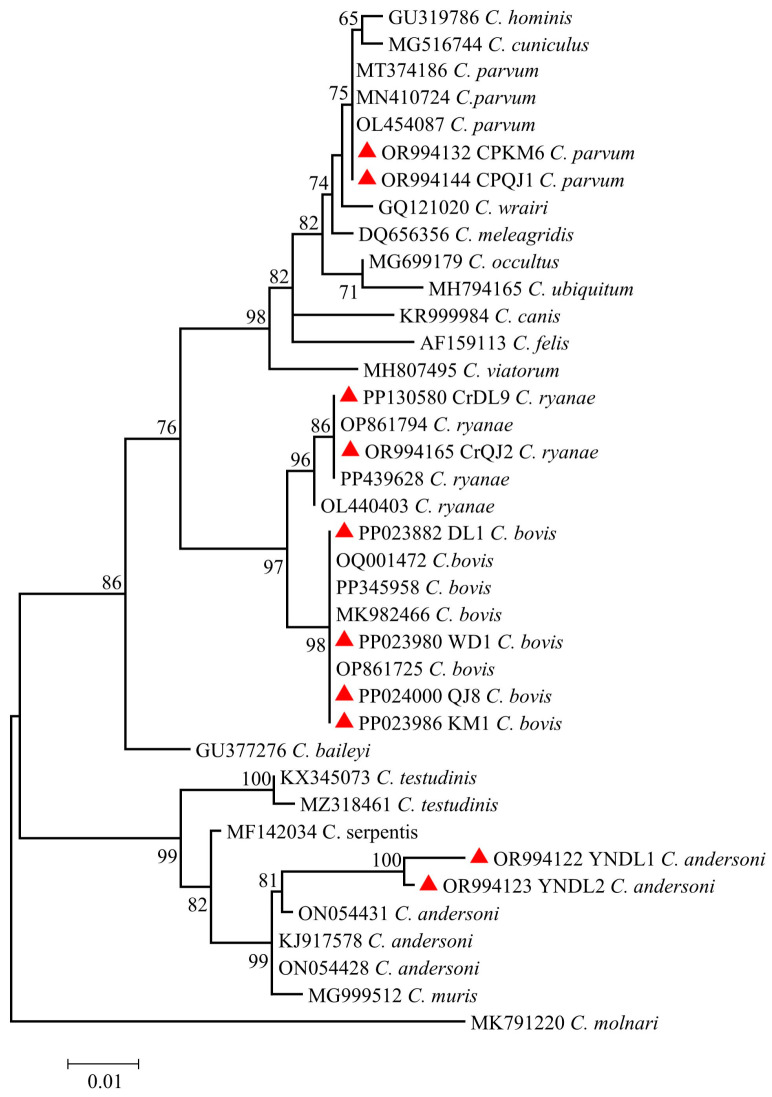
The phylogenetic relationship of *Cryptosporidium* spp. based on the *SSU* rRNA gene. 🔺: The *SSU* rRNA gene sequence of *Cryptosporidium* spp. obtained in this study.

The nucleotide sequences of *C. bovis*, *C. parvum*, *C. ryanae* and *C. andersoni* obtained in this study were identical to the reference sequences OQ001472 (*C. bovis*), OL454087 (*C. parvum*), OP861794 (*C. ryanae*) and ON054431 (*C. andersoni*), respectively. Partial sequences were selected to construct a phylogenetic evolutionary tree (Figure 3). The sequences obtained in this study have been deposited in GenBank under the accession numbers PP023882-PP024000 and OR994122-OR994166.

### 3.3. Subtyping of Cryptosporidium spp.

In this study, *C. bovis*, *C. parvum* and *C. ryanae* were subtyped based on the *gp60* locus. A total of 60 out of 119 *C. bovis*-positive samples were subtyped into six genetic groups, namely XXVIa (*n* = 3), XXVIb (*n* = 26), XXVIc (*n* = 3), XXVId (*n* = 3), XXVIe (*n* = 18) and XXVIf (*n* = 7) (Table 2). A total of 12 out of 23 *C. parvum* samples were successfully typed, and two subtypes belonging to the IId family were detected: IIdA19G1 (*n* = 7) and IIdA18G1 (*n* = 5) (Table 2). Only 5 out of 20 *C. ryanae*-positive samples were identified, with 4 subtypes detected: XXId (*n* = 1), XXIe (*n* = 1), XXIf (*n* = 2) and XXIg (*n* = 1) (Table 2). Two cases of *C. andersoni*-positive samples were subtyped by using MLST at four loci (MS1, MS2, MS3 and MS16) but were unsuccessful.

## 4. Discussion

A high occurrence of *Cryptosporidium* spp. was detected in pre-/post-weaned calves in Yunnan Province, southwest China. The overall prevalence of *Cryptosporidium* was 32.9% (164/498), which was similar to the global pooled incidence of 29.1% for bovine cryptosporidiosis [44], but higher than that in dairy cattle in China (13.9% [6], 10.4% [16] or 17.0% [45]). The infection rate observed in the current study was close to that in northeastern China (29.8%) and higher than that in central China (16.9%), eastern China (17.4%), northern China (15.7%), northwestern China (15.8%), southern China (9.5%) and southwestern China (13.7%) [45]. Compared with the prevalence of *Cryptosporidium* infection in dairy cattle in other provinces of China, the infection rate in this study was lower than that in Xinjiang (48.7%) [39], Henan (36.2%) [46] and Shanghai (37.0%) [47], but higher than that in the other remaining provinces [30,38,48,49,50,51,52,53,54,55]. It was also higher than the rate reported in only two surveys in Yunnan [35,41]. This result was expected, because young animals are more susceptible to *Cryptosporidium* infection on farms. Also, the overall prevalence detected in our study was higher than that in Malaysia (15.1%) [56], Canada (15.4%) [57], Korea (18.6%) [58] and Thailand (13.5%) [59], but lower than that in other countries, such as Germany (88.9%) [60], Spain (57.8%) [61], Brazil (52.9%) [62], Italy (38.8%) [63] and Egypt (33.5%) [64].

In this study, regional differences in detection rates of *Cryptosporidium* spp. were observed. The *Cryptosporidium* prevalence ranged from 12.7% to 45.3% in the four sampling regions. The difference in infection rate among different regions was extremely significant (*p* < 0.01), which was consistent with previous studies showing that infection rates varied significantly for different regions/provinces in China [16]. The infection rate of *Cryptosporidium* species was also higher in female cattle than in male cattle, although the difference was not statistically significant (*p* = 0.63), which was consistent with previous findings [35,65,66]. In contrast, other studies reported a significant role of gender in the prevalence of *Cryptosporidium* in calves [67,68]. In addition, the prevalence of *Cryptosporidium* in dairy cattle in the current study was different from that in other cattle breeds in China [40,69], such as yak (10.5%), beef cattle (10.4%) and buffalo (15.5%). However, it was difficult to compare the prevalence data, as they were influenced by various factors, including livestock production systems, breeds, sample sizes, geographical differences, diagnostic methods, ecology and seasons.

Four *Cryptosporidium* species were detected in this study, and these have been reported in cattle worldwide [10,17] and also in China [16]. In the current study, calves in Yunnan were mainly found to be infected with *C. bovis* (72.5%), followed by *C. parvum* (14.6%), *C. ryanae* (12.2%) and *C. andersoni* (1.2%). The distribution of *Cryptosporidium* species in young dairy calves was reported to be different between small farms and concentrated animal-feeding operations (CAFOs) [70]. *C. parvum* is more common in pre-weaned calves in many industrialized countries [21] as well as in some regions of China [36,39,53,71], but this did not seem to be the case in our study, where *C. bovis* was the predominant species in pre- and post-weaned calves in Yunnan Province. Our result is consistent with Guangdong [19], Hubei [30], Jiangxi [34], Sichuan [38] and Gansu [50].This may be related to differences in farming practices and scale, as the farms we sampled were small-scale farms rather than CAFOs. In this study, weaned calves were predominantly infected with *C. bovis* (70.6%), which was also found in some farms in India, Japan, Sweden, Vietnam and the USA [22,72,73,74,75,76]. As noted in previous reports, *C. ryanae* infections appeared later than those caused by *C. parvum* and *C. bovis* [18]. Only two cases of *C. andersoni* infection were identified in this study. This result is expected, as *C. andersoni* is usually found in yearlings and adults [20].

A high diversity of *C. bovis* and *C. ryanae* was detected in this study. Six *C. bovis* subtype families, XXVIa to XXVIf, were identified, which was consistent with Shanghai and Guangdong, rather than Hunan, Jiangsu, Heilongjiang and Henan [26]. A previous study showed that there were five subtype families in Yunnan (XXVIa, XXVIb, XXVIc, XXVId and XXVIf), in which XXVIe was absent, and the dominant subtype was XXVIa [26]. Notably, in contrast to other studies, XXVIe was the dominant subtype in our analysis. Although *C. bovis* does not infect humans, it is detected mainly in young calves and is associated with the occurrence of moderate diarrhea, leading to production losses [12,18,33]. Eight subtype families of *C. ryanae* have been observed in dairy cattle [27], namely XXIa to XXIh. Four subtypes (XXId, XXIe, XXIf and XXIg) were identified in this study, but XXIa, which is the dominant subtype family in dairy cattle, was not found [27]. Instead, XXIe and XXIf appeared to be more common. Compared with Heilongjiang, Hebei, Shanghai and Guangxi, the genetic diversity of *C. ryanae* in Yunnan was higher, but it was lower than in Guangdong [27]. These differences may be related to differences in geography, feeding models and sanitary conditions. Unfortunately, two *C. andersoni* cases in our investigation were not properly subtyped, which may be due to the fact that its main infection hosts are young and adult cattle, resulting in fewer oocysts in the feces of calves.

Two *C. parvum* subtypes (IIdA18G1 and IIdA19G1) were identified in this study. The results of this study also have public health implications, since some subtypes detected in *C. parvum* have previously been identified in human samples. *C. parvum* is considered the most important zoonotic species, causing human cryptosporidiosis, severe watery diarrhea and the death of newborn calves [5]. Both IIa and IId are zoonotic subtype families [15], and the IId subtypes are exclusively found in dairy cattle in China [18]. The prevalence of *C. parvum* infection in dairy cattle in China has dramatically increased in recent years [77]. To date, seven subtypes (IIdA14G1, IIdA15G1, IIdA17G1, IIdA19G1, IIdA20G1, IIdA21G1 and IIdA24G2) of *C. parvum* have been observed in dairy cattle in China [17,36]. IIdA19G1 is widely distributed in dairy cattle in many provinces, such as Hebei [32], Heilongjiang [36], Xinjiang [52], Shaanxi [78], Shanghai [47], Beijing [53], Guangdong [19] and Gansu [37]. In addition, the IIdA19G1 subtype caused a *Cryptosporidium* outbreak in neonatal calves with a mortality rate of approximately 60% on a dairy farm in Jiangsu Province, China, indicating that this subtype is highly virulent [12]. Notably, the *C. parvum* IIdA19G1 subtype has also been identified in AIDS patients [79], suggesting a high zoonotic potential in animals and humans. In this study, the IIdA18G1 subtype was found to be one of the dominant subtypes in cattle in Yunnan Province, which may lead to an increased likelihood of cryptosporidiosis outbreaks in dairy cattle. The IIdA18G1 subtype has previously been reported to occur in calves in Serbia and Montenegro [80], Turkey [81] and Sudan [82], in yaks and sheep in China [83], and in humans in Qatar [84], the United Kingdom [85], Spain [86] and Kuwait [87], whereas only one case was reported in dairy cattle in Yunnan Province, China [41]. So far, IIdA18G1 has only been detected in cattle in one case report in Yunnan, and the absence of this subtype in other regions of China suggests its endemicity, as it has not been observed to spread geographically. However, both IIdA18G1 and IIdA19G1 are well-known human pathogens, indicating that zoonotic *Cryptosporidium* could pose a potential threat to public health, and further efforts are needed to monitor the prevalence and molecular characterization of *C. parvum* in dairy cattle in Yunnan.

## 5. Conclusions

This study presented a high incidence of *Cryptosporidium* spp. infection in Holstein calves in Yunnan Province, adding to the scarce data on the distribution of *Cryptosporidium* in dairy cattle in Yunnan. This will improve our knowledge of cryptosporidiosis epidemiology and how to evaluate the potential threat of *Cryptosporidium* infections in cattle to human health. Four different *Cryptosporidium* species were identified, namely *C. bovis*, *C. parvum*, *C. andersoni* and *C. ryanae*. The results of the present study show that the dominant species of *Cryptosporidium* spp was *C. bovis*, and six subtypes (XXVIe, XXVIb, XXVIf, XXVIa, XXVIc and XXVId) of *C. bovis*, four subtypes (XXIf, XXId, XXIe and XXIg) of *C. ryanae* and two zoonotic subtypes (IIdA19G1 and IIdA18G1) of *C. parvum* were identified, highlighting the diversity of this pathogen in the study area. It is worth noting that the IIdA18G1 and IIdA19G1 subtypes of *C. parvum* pose an increasing threat to human health. Therefore, it is urged to apply effective measures and practices to prevent and control *Cryptosporidium* infection in dairy cattle in order to reduce or eliminate its potential threat to public health.

## Figures and Tables

**Table 1:** Occurrence and factors associated with *Cryptosporidium* spp. in Holstein cattle in Yunnan Province, China.

Variable	Category	Sample Size	No. Positive	Prevalence (%) (95%CI)	OR (95%CI)	*p*-Value
Region	Kunming	53	24	45.3 (31.43–59.13)	5.68 (2.60–12.37)	<0.01
	Dali	312	120	38.5 (33.00–43.90)	4.29 (2.34–7.85)	
	Chuxiong	23	6	26.1 (6.67–45.50)	2.42 (0.82–7.17)	
	Qujing	110	14	12.7 (6.40–19.05)	Reference	
Sex	Female	392	127	32.4 (27.74–37.05)	Reference	0.63
	Male	106	37	34.9 (25.68–44.13)	1.12 (0.71–1.76)	
Age	Pre-weaned (0–60 days)	316	106	33.5 (28.31–38.78)	1.08 (0.73–1.59)	0.70
	Post-weaned (61–180 days)	182	58	31.9 (25.03–38.70)	Reference	
Total		498	164	32.9 (28.79–37.07)		

No.: number; CI: confidence interval; OR: odds ratio.

**Table 2:** Subtypes and factors associated with *Cryptosporidium* spp. in Holstein cattle in Yunnan Province, China.

Factor	Category	*Cryptosporidium* Species	*Cryptosporidium* Subtypes		
			*C. bovis*	*C. parvum*	*C. ryanae*
Region	Dali	*C. bovis* (98), *C. ryanae* (17), *C. parvum* (3), *C. andersoni* (2)	XXVIb (22), XXVIe (17), XXVIf (7), XXVId (3), XXVIc (2) , XXVIa (1)	–	XXIf (2), XXId (1), XXIe (1), XXIg (1)
	Kunming	*C. parvum* (17), *C. bovis* (7)	XXVIb (4), XXVIa (1)	IIdA19G1(7), IIdA18G1 (3)	–
	Qujing	*C. bovis* (8), *C. parvum* (3), *C. ryanae* (3)	XXVIa (1)	IIdA18G1 (2)	–
	Chuxiong	*C. bovis* (6)	XXVIb (18),XXVIc (1), XXVIe (1)	–	
Sex	Female	*C. bovis* (84), *C. parvum* (23), *C. ryanae* (18), *C. andersoni* (2)	XXVIe (15), XXVIa (3), XXVIc (3), XXVId (2), XXVIf (1)	IIdA19G1 (7), IIdA18G1 (5)	XXIf (2), XXIe (1), XXIg (1)
	Male	*C. bovis* (35), *C. ryanae* (2)	XXVIb(8), XXVIf (6), XXVIe (3), XXVId (1)	–	XXId (1)
Age	Pre-weaned (0–60 days)	*C. bovis* (78), *C. parvum* (22), *C. ryanae* (5), *C. andersoni* (1)	XXVIb (20), XXVIe (14), XXVIf (4), XXVIa (3), XXVIc (1), XXVId (1)	IIdA19G1 (7), IIdA18G1 (5)	XXId (1), XXIf (1)
	Post-weaned (61–180 days)	*C. bovis* (41), *C. ryanae* (15), *C. andersoni* (1), *C. parvum* (1)	XXVIb (6), XXVIe (4), XXVIf (3), XXVIc (2), XXVId (2)	–	XXIe (1), XXIf (1), XXIg (1)
Total		*C. bovis* (119), *C. parvum* (23), *C. ryanae* (20), *C. andersoni* (2)	XXVIb (26), XXVIe (18), XXVIf (7), XXVIa (3), XXVIc (3), XXVId (3)	IIdA19G1 (7), IIdA18G1 (5)	XXIf(2), XXId (1), XXIe (1), XXIg (1)

Note: “–” indicates absence.

## Data Availability

The data that support the findings are in the possession of the authors. The data of this study are available on request from the corresponding author.

## Supplementary Materials

The following supporting information can be downloaded at: https://www.mdpi.com/article/10.3390/ani14131907/s1, Table S1: Primers and expected amplicon sizes for PCR amplification.
