# Supplementary material for: Cryptosporidium spp. Infection and Genotype Identification in Pre-Weaned and Post-Weaned Calves in Yunnan Province, China
Source: Animals (Basel). 2024 Jun 27;14(13):1907. doi: 10.3390/ani14131907 (PMC11240314; doi:10.3390/ani14131907)
Supplement: Supplementary file 1 [file animals-14-01907-s001.zip › animals-3029785-supplementary.pdf]

Supplementary Material

Cryptosporidium spp. infection and genotype identification in pre-weaned and post-weaned calves in Yunnan Province, China

Meng-Ling Deng[1,2†], Zhao-Jun Heng[2†], Liu-Jia Li[3], Jian-Fa Yang[1,2], Jun-Jun He[2], Feng-Cai Zou[1,2*], Fan-Fan Shu[2*]

[1]Faculty of Animal Science and Technology, Yunnan Agricultural University, Kunming, Yunnan Province 650201, People's Republic of China
[2]Key Laboratory of Veterinary Public Health of Yunnan Province, College of Veterinary Medicine, Yunnan Agricultural University, Kunming, Yunnan Province 650201, People's Republic of China
[3]College of Agriculture and Biological Science, Dali University, Dali, Yunnan Province 671003, People's Republic of China
* Correspondence: zfc1207@vip.163.com (F.-C.Z.); shuff1227@163.com (F.-F.S.)
† These authors contributed to the research equally.

Table S1. Primers and expected amplicon sizes for PCR amplification.

Gene	Primers (Squence 5′-3′)	Annealing temperatures (°C)	Fragment lengths (bp)
SSU rRNA	TTC TAG AGC TAA TAC ATG CG (F1)	55	1320
	CCC ATT TCC TTC GAA ACA GGA (R1)		
	GGA AGG GTT GTA TTT ATTA GAT AAA G (F2)	58	830
	CCC ATT TCC TTC GAA ACA GGA (R2)		
gp60	TTA CTC TCC GTT ATA GTC TCC (F1)	55	850
(*C. parvum*)	GGA AGG AAC GAT GTA TCT GA (R1)		
	TCC GCT GTA TTC TCA GCC (F2)	58	
	GCA GAG GAA CCA GCA TC (R2)		
gp60	ATG CGA CTT ACG CTC TAC ATT ACT CT (F1)	55	1300
(*C. bovis*)	GAC AAA ATG AAG GCT GAG ATG ATG GGA (R1)		
	CCT CTC GGC ATT TAT TGC CCT (F2)	55	
	ATA CCT AAG GCC AAA TGC TGA TGA A (R2)		
gp60	GCT CGA GTT CTG AGT CGA (F1)	55	1024
(*C. ryane*)	ATA CCG TTA AAA TGA AGG CCA A (R1)		
	CCT CAG ATA ATG AGC AGT CTA (F2)	55	
	GAT GGG ATA ACA TAT CTA TAA CCA AA (R2)		
MS1	ACC ATC TAG AGA TAA CGA GCG A (F1)	55	550
(*C. andersoni*)	GAA TCA GAA GAT GAG CGA CAA (R1)		
	CGT GAT AGT GGG TAT GAA TTG GAC A (F2)	55	
	CGA CTG CGA TAC TCA CGT CCT (R2)		
MS2	TTG CAA CTG TAC CTA AAT TAG TA (F1)	55	457
(*C. andersoni*)	GTG AGA CTT CTG GGG TCC TGA (R1)		

	TCA TGA CGC GTC ATA CCA ACA (F2)	52	
	ACT TAG ACA GTT CTA TGC TGA (R2)		
MS3	AAC CAA GTG AAT CAC GAA CTT (F1)	55	536
(*C. andersoni*)	TCA AGT ACA GCA GTC TAT TGC TT (R1)		
	GCA ATA TCT TCG ACG ATC CCA (F2)	55	
	ATG GGA ATA ATT CTT CAT CAT CAA (R2)		
MS16	GAA GAG GTC GAA GTT AAG CTA (F1)	55	597
(*C. andersoni*)	GAC AAT CAT CTA AAT CGT GTT (R1)		
	AAG TTT CAT CTA GGT ACA CTA AGA (F2)	50	
	CAC TAC CTA ATC TCG TGT ACT T (R2)		
